# Clinical and laboratorial profile and histological features on minor salivary glands from patients under investigation for Sjögren’s syndrome

**DOI:** 10.4317/medoral.19486

**Published:** 2013-12-07

**Authors:** Débora L. Pereira, Verônica S. Vilela, Teresa C R B. dos-Santos, Fábio R. Pires

**Affiliations:** 1 DDS, Trainée, Oral Pathology, School of Dentistry, State University of Rio de Janeiro, Rio de Janeiro, Brazil; 2 MD, Rheumatology, Pedro Ernesto University Hospital, State University of Rio de Janeiro, Rio de Janeiro, Brazil; 3 DDS, MSc, Assistant professor, Oral Pathology, School of Dentistry, State University of Rio de Janeiro, Rio de Janeiro, Brazil; 4 DDS, PhD, Adjunct professor, Oral Pathology, School of Dentistry, State University of Rio de Janeiro, Rio de Janeiro, Brazil

## Abstract

Diagnosis of Sjögren’s syndrome (SS) is complex and the usefulness of labial minor salivary glands biopsy in this process remains controversial. Objectives: to evaluate the clinical and laboratorial profile and histological features on labial minor salivary glands from patients under investigation of SS. Study Design: clinical charts from 38 patients under suspicion of SS and submitted to labial minor salivary glands biopsies were reviewed. Clinical and laboratorial data were retrieved from the clinical files and the HE-stained histological slides were reviewed under light microscopy. Results: mean age of the patients was 56.5 years and 97% were females; histological analysis showed that 42% of the cases showed ductal dilatation, lymphocytic foci were found in 52.6% and, from this group, 80% of the cases presented a foci/lobules ratio above 0.8. Acinar/ductal ratio was considered diminished in 39.5% of the samples. Thirty six (95%) and 32 (84%) patients, respectively, complained about xerostomia and xerophthalmia. A study of the time interval of the symptoms that led to SS investigation showed a mean of 116 months. Moreover, sixty-six percent of the patients had already been submitted to immunosuppressive therapy prior to the labial minor salivary gland biopsy. Age of the patients, scintigraphic alterations on salivary function, antinuclear factor (ANF), anti-Ro and anti-La did not show statistical significant association with the histological features. Lobules/foci ratio above 0.8 was the only histological parameter statistically associated with Sjögren’s syndrome diagnosis (*p*<0.0001). Conclusions: in the studied sample, lymphocytic foci on salivary glands were the only histological parameter associated to the diagnosis of SS. Early indication of labial minor salivary gland biopsy to patients under investigation of SS could limit the effects of immunosuppressive therapy on the histological features associated with the evolution of salivary gland involvement in SS.

** Key words:**Sjögren syndrome, minor salivary glands, biopsy, lymphocytic foci.

## Introduction

Sjögren’s syndrome (SS) is a chronic autoimmune disease characterized by xerophthalmia and xerostomia, functional alterations in both salivary and lacrimal glands and the presence of specific and unspecific serum autoantibodies in the affected patients (1-4). Diagnosis of SS is complex and depends on subjective symptoms and clinical, serological and histological features, obtained through investigations performed by a multidisciplinary team, including rheumatologists, ophthalmologists, clinicians and dentists (5-7). Biopsy of labial minor salivary glands has been considered a diagnostic adjunctive technique with high specificity for SS, with few post surgical complications (8-10). Nevertheless, its regular use on SS diagnosis is not a routine at some reference centers, especially due to the need for careful histological evaluation of the specimens and the invasive nature of the procedure. The aim of the present study was to retrospectively evaluate the clinical and laboratorial parameters and the detailed histological features of labial minor salivary glands from a group of patients under investigation for SS.

## Material and Methods

All specimens from biopsies directed to the removal of labial minor salivary glands performed in patients under investigation for Sjögren’s syndrome registered in the Oral Pathology laboratory, School of Dentistry, State University of Rio de Janeiro, from 2006 to 2009 were reviewed. Histological slides containing hematoxylin and eosin (HE)-stained 5µm sections were analyzed under light microscopy (Eclipse E-200 microscope, Nikon, Japan), according to the following criteria: number of salivary gland lobules; percentage of adipose tissue on the salivary glands (less than 10% or more than 10%); absence or presence of ductal dilatation, acinar atrophy and sclerosis of the glandular connective tissue; acinar/ductal ratio (normal ratio, similar to normal glands; reduced ratio, with increased ductal component); the number of lymphocytic foci (1 focus was composed of at least 50 grouped lymphocytes); and the ratio number of lobules/number of foci. The established ratio as the limit between the groups considered “compatible” and “non-compatible” with the diagnosis of Sjögren’s syndrome was 0.8, which means the presence of at least 5 lymphocytic foci in 4 minor salivary gland lobules. This criterion was used in addition to the absolute number of lymphocytic foci due to the fact that in some cases the submitted specimens were constituted by less than 5 salivary gland lobules.

After selection and review of all HE-stained histological slides, clinical and laboratory information was retrieved from the clinical records of all patients on the Rheumatology service at Pedro Ernesto University Hospital, School of Medicine, State University of Rio de Janeiro. Data included age, gender, symptoms of xerostomia and xerophthalmia, results from the Schirmer tests and major salivary gland scintigraphy, history of recurrent parotitis, pulmonary involvement and presence of other concomitant rheumatoid autoimmune diseases. Serum laboratory data retrieved from the clinical records included autoantibodies, such as rheumatoid factor (RF), ANF, anti-Ro and anti-La. Time interval with the symptoms that led to the investigation of Sjögren’s syndrome and history of previous/present immunosuppressive treatment before the labial minor salivary gland biopsies were also retrieved. Patients treated or under follow-up in other institutions, without clinical records presenting sufficient data for analysis, and showing HE-stained slides presenting no sufficient available tissue for histological analysis were excluded from the final studied sample. Data were tabulated and analyzed with the aid of the Statistical Package for Social Science (SPSS version 8.0, SPSS Inc., Chicago, Illinois, United States, 1997) with statistical significance level of 5% (*p*<0.05). This study was approved by the Ethics Committee, State University of Rio de Janeiro (COEP 059/2010).

## Results

**Figure 1:**
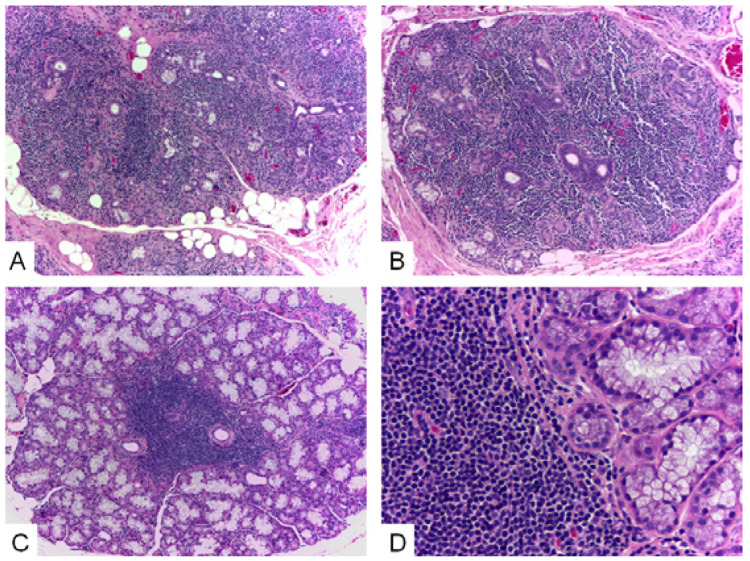
A-B) Minor salivary glands showing extensive lymphocytic infiltration, destruction of the acinar component and preservation of the ductal structures (HE, 10x); C) Focal lymphocytic infiltration characteristic of Sjögren’s syndrome (HE, 10x); D) Detail of the limit between the lymphocytic infiltrate and the preserved minor salivary gland acini (HE, 40x).

Seventy-seven HE-stained histological slides containing labial minor salivary glands from patients under investigation of Sjögren’s syndrome were reviewed. From this initial sample, 38 cases presenting sufficient clinical and laboratory information were selected, representing the final studied sample. Patients from this group presented a mean age of 56.5 years (ranging from 30 to 78 years) and 37 patients were females (97%). Histological analysis showed that 55.3% presented more than 10% of glandular adipose tissue and 42% of the cases showed ductal dilatation. Acinar atrophy and connective tissue sclerosis were found in 15.8% and 10.5% of the cases, respectively. Lymphocytic foci were found in 52.6% of the cases and, from this group, 80% of the cases presented a foci/lobules ratio above 0.8. Acinar/ductal ratio was considered diminished in 39.5% of the samples. Figure 1 (A-D) shows some histological features from the affected minor salivary glands.

Thirty six (95%) and 32 (84%) patients, respectively, complained about xerostomia and xerophthalmia. Schirmer test was performed in 29 patients (76%), of which 18 (62%) showed results compatible with Sjögren’s syndrome. Eight out of the 38 patients (21%) reported previous episodes of recurrent parotitis and 20 out of the 33 patients (61%) submitted to salivary gland scintigraphy showed altered salivary function. Thirty two patients (84%) reported being diagnosed with other autoimmune diseases, especially rheumatoid arthritis, lupus erythematosus and fibromyalgia, and 5 patients (13%) reported associated pulmonary symptoms. Laboratory exams showed that out of the 32 patients tested for ANF, 17 (53%) showed positive results. In contrast, from the 24 patients who had been tested for RF, only 4 (17%) showed positive results. Anti-Ro and anti-La were requested together for 24 patients and 9 (38%) showed positive results for both. Time interval with the symptoms that led to Sjögren’s syndrome investigation ranged from 1 to 288 months, with a mean of 116 months, and 25 out of the 38 patients (66%) had already been submitted to immunosuppressive therapy prior to labial minor salivary gland biopsy. Time interval from the beginning of the symptoms and the labial minor salivary gland biopsy ranged from 4 to 312 months, with a mean of 91 months.

 Table 1 shows the distribution of the histological parameters according to age, results from the salivary gland scintigraphy and laboratory parameters from the studied patients. Age of the patients, scintigraphic alterations on salivary function, ANF, anti-Ro and anti-La did not show statistical significant association with the histological features. Although only 4 (17%) out of the 24 patients tested for RF presented positive results, all showed reduced acinar/ductal ration and lobules/foci ratio over 0.8 ( Table 1). It was also observed that 12 out of the 25 patients (48%) under use of immunosuppressant prior to labial biopsy showed a lobules/foci ratio over 0.8 and reduced acinar/ductal ratio. One third of the patients with negative results to anti-Ro and anti-La showed a lobules/foci ratio over 0.8, but the presence of anti-Ro and anti-La did not show statistical significant association with the presence of lymphocytic foci (p=0.657 and 0.343, respectively).

**Table 1:**
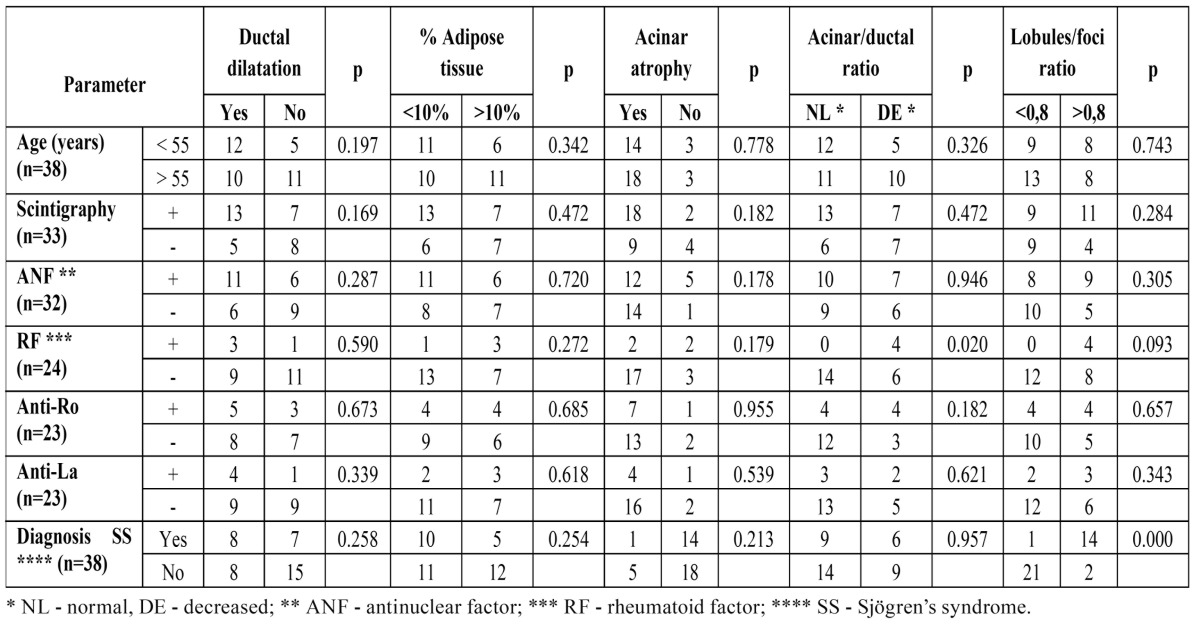
Distribution of the histological features observed on labial minor salivary glands according to clinical and laboratorial parameters.

Lobules/foci ratio above 0.8 was the only histological parameter statistically associated with Sjögren’s syndrome diagnosis (*p*<0.0001). Out of the 38 patients, 16 (42%) showed lobules/foci ratio above 0.8, out of which 14 were diagnosed with Sjögren’s syndrome after analysis of other criteria. One patient presented a lobules/foci ratio lower than 0.8, but it was diagnosed with the syndrome due to the presence of other clinical and laboratorial parameters. Two patients were not diagnosed with Sjögren’s syndrome, despite presenting a lobules/foci ratio above 0.8, due to lack of confirmation after analysis of the other parameters ( Table 1).

## Discussion

SS is a complex disease and diagnosis can be challenging in many cases. Depending on the country/region of origin of the study, SS can affect up 0.3 to 0.6% of the general population, with predilection for adult females on their 5th to 6th decades of life (2,3). It can be diagnosed as an isolated disease (primary SS) or associated to other autoimmune diseases, such as rheumatoid arthritis and lupus erythematosus (associated SS) (11,12). SS etiology remains unknown, but combined genetic and environmental factors seem to participate in its pathogenesis (2). Several clinical, laboratorial, imaginological and histological parameters have proved to be useful in SS diagnosis, and two different classification/diagnosis systems are currently being used for the disease, the American-European Consensus Group (13) and the Sjögren’s International Collaborative Clinical Alliance (14). Both have considered labial minor salivary gland biopsy as a useful tool for SS diagnosis, due to the possibility of confirming the glandular damage and disease activity directly on the affected tissue. The present study focused on evaluating the association of clinical and laboratorial parameters with histological features in a subset of patients under investigation of SS. As expected, the profile of the sample was composed of adult females, reinforcing the possibility that hormonal and genetic alterations can, at least partially, modulate the development of the disease (7).

Daniels *et al*. have recently reinforced the importance of labial minor salivary gland biopsy for SS diagnosis, due to its high specificity, allowing the analysis of disease activity directly on the target organ (9). Although some authors have considered labial minor salivary gland biopsy an invasive procedure, intraoperative problems and post-surgical sequelae are unusual (15). The most important limitation of the technique is the difficulty in establishing definite and objective histological diagnostic criteria capable of adequately characterize the specimens associated with SS. The pathologist’s individual subjective analysis can reveal considerable variability, even knowing that the presence of lymphocytic foci is considered the gold standard for SS diagnosis (9,16,17). Critical analysis of the detailed histological parameters is essential for the evaluation of their usefulness in SS diagnosis (18). The present study focused on the evaluation of some histological parameters observed in minor salivary glands derived from labial biopsies for SS diagnosis, but lymphocytic foci remained as the only statistically significant histological feature. It was interesting to notice that all patients presenting positive RF showed a reduced acinar/ductal ratio and lobules/foci ratio above 0.8, but the low number of cases precludes any significance of these results. Analysis of a larger sample focusing on this parameter could offer new insights on this topic.

Another limitation of the affected salivary glands analysis is associated with the large time interval from the beginning of the symptoms and the biopsy. In the present study the time interval from the first symptoms and the biopsy showed a mean of 91 months, reinforcing this problem. Patients usually start immunosuppressive therapy aimed at the control of the main symptoms and complaints associated with the disease (especially in associated SS) during this time interval, and it is believed that some histological parameters could be altered by local and systemic effect of the drugs. Zandbelt *et al*., have reported alterations on the histological parameters in SS-affected minor salivary glands before and after corticosteroid therapy, additionally suggesting that labial minor salivary gland biopsy can be useful in monitoring activity of the disease (19). The results of the present study showed that most patients had been submitted to or were on immunosuppressive therapy when the biopsies were performed. Even so, almost half the patients presented reduced acinar/ductal ratio and lobules/foci ratio above 0.8, fulfilling the histological criteria of SS. It is possible to speculate that these values would have been higher if it were not for the interference of the immunosuppressive therapy.

The importance of serological autoantibodies, such as anti-Ro and anti-La, on SS diagnosis has been demonstrated and, in the clinical practice, serological exams usually precede labial minor salivary gland biopsy on routine SS diagnosis (13,15,20). The results of the present study showed that more than 50% of the cases tested for anti-Ro and anti-La presented negative results. However, about one third of such cases presented histological lymphocytic foci compatible with SS. This data reveals that without minor salivary gland biopsy, a high percentage of patients would remain underdiagnosed due to the lack of immunological criteria. These results are similar to the results from Langerman *et al*., which showed that only 53% of the patients who presented serological positive results showed lymphocytic foci compatible with SS (18).

The most representative histological feature associated with SS is the presence of lymphocytic foci. Bookman *et al*., have recently demonstrated the correlation of glandular fibrosis and reduction on salivary flow in SS affected patients (21). In the samples analyzed in the present study, minor salivary gland connective tissue sclerosis as well as acinar atrophy, were found in solely 15.8% and 10.5% of the cases, respectively, restricting their correlation with the other parameters. It is important to point out that these histological characteristics carry a variable subjective component during evaluation, limiting their routine use as an auxiliary parameter on SS diagnosis and progression.

A large recent multicentric study including clinical and histological data from 1726 subjects have demonstrated a strong association between the presence of lymphocytic foci on the affected minor salivary glands and serological parameters (higher titers of ANF, RF, anti-Ro and anti-La) and a weak correlation with subjective parameters, such as xerostomia and xerophthalmia (9). The results from the present study showed no association of the clinical, serological and histological studied parameters. However, the limited number of cases and the long time interval for the indication of the labial minor salivary gland biopsy in some cases under SS investigation may have modulated the results.

In the studied sample, lymphocytic foci on salivary glands were the only histological parameter associated to the diagnosis of SS. Early indication of labial minor salivary gland biopsy to patients under investigation of SS could limit the effects of immunosuppressive therapy on the histological features associated with the evolution of salivary gland involvement in SS.
